# Low dimensional nanostructures of fast ion conducting lithium nitride

**DOI:** 10.1038/s41467-020-17951-6

**Published:** 2020-09-08

**Authors:** Nuria Tapia-Ruiz, Alexandra G. Gordon, Catherine M. Jewell, Hannah K. Edwards, Charles W. Dunnill, James M. Blackman, Colin P. Snape, Paul D. Brown, Ian MacLaren, Matteo Baldoni, Elena Besley, Jeremy J. Titman, Duncan H. Gregory

**Affiliations:** 1 WestCHEM, School of Chemistry, University of Glasgow, Glasgow, G12 8QQ UK; 2 School of Chemistry, University of Nottingham, University Park, Nottingham, NG7 2RD UK; 3 Department of Mechanical, Materials and Manufacturing Engineering, University of Nottingham, University Park, Nottingham, NG7 2RD UK; 4 Department of Chemical and Environmental Engineering, University of Nottingham, University Park, Nottingham, NG7 2RD UK; 5 School of Physics and Astronomy, University of Glasgow, Glasgow, G12 8QQ UK; 6 Istituto per lo Studio dei Materiali Nanostrutturati (ISMN), Consiglio Nazionale delle Ricerche (CNR), Via P. Gobetti 101, 40129 Bologna, Italy; 7 Present Address: Department of Chemistry, Lancaster University, Lancaster, LA1 4YB UK

**Keywords:** Chemistry, Materials science

## Abstract

As the only stable binary compound formed between an alkali metal and nitrogen, lithium nitride possesses remarkable properties and is a model material for energy applications involving the transport of lithium ions. Following a materials design principle drawn from broad structural analogies to hexagonal graphene and boron nitride, we demonstrate that such low dimensional structures can also be formed from an s-block element and nitrogen. Both one- and two-dimensional nanostructures of lithium nitride, Li_3_N, can be grown despite the absence of an equivalent van der Waals gap. Lithium-ion diffusion is enhanced compared to the bulk compound, yielding materials with exceptional ionic mobility. Li_3_N demonstrates the conceptual assembly of ionic inorganic nanostructures from monolayers without the requirement of a van der Waals gap. Computational studies reveal an electronic structure mediated by the number of Li-N layers, with a transition from a bulk narrow-bandgap semiconductor to a metal at the nanoscale.

## Abstract (web summary)

Lithium nitride is the only stable binary alkali metal-nitrogen compound and shows promise for energy applications involving the transport of lithium ions. Here, the authors demonstrate that lithium nitride nanostructures can be grown as fibres and sheets despite the absence of a van der Waals gap.

## Introduction

Lithium nitride, Li_3_N, was originally proposed for use as an electrolyte in all solid-state Li^+^ ion batteries given its exceptional ionic conductivity at room temperature (ca. 10^−3^ S cm^−1^)^1^. Indeed, for several decades it remained the highest conducting crystalline Li^+^ ion conductor at ambient conditions hampered chiefly by its low decomposition potential despite many ongoing attempts to stabilise it. Doping with late transition metals, however, triggers electronic conductivity that can be exploited in anodes with more than twice the charging capacity of graphite^2^. Li_3_N has also been proposed for a myriad of other applications, e.g. as a means of converting CO_2_ into useful products^3^, as the electron injection layer in organic light-emitting diodes^4^ and as an unusual reducing agent in preparative organic and organometallic chemistry^5^. Further, in 2002 Li_3_N was revealed as a potential candidate for solid-state hydrogen storage given its capability to accommodate up to 10.4 wt.% H_2_^6^. Slow kinetics for H_2_ sorption and high (de)hydrogenation temperatures are the primary hurdles to overcome before the Li–N–H system can be exploited commercially, however. By combining experiment and calculation, we demonstrate how the changes in electronic structure and reduction of diffusion lengths brought about by chemically nanostructuring Li_3_N can lead to dramatic changes in electronic properties and ionic transport behaviour.

## Results

### Synthesis and characterisation

**Fig. 1:**
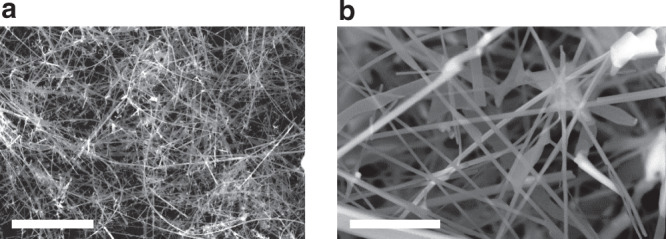
SEM micrographs of typical Li_3_N nanofibres (type I) at different magnifications. Images are taken at: **a** ×500 and **b** ×5000. Scale bars correspond to 50 μm (**a**) and 5 μm (**b**).

Li_3_N nanocrystals grow following the heating and cooling of the bulk nitride powder under a reduced pressure of nitrogen (see “Methods”). Powder X-ray diffraction (PXD) data for the fine powder product matches to hexagonal α-Li_3_N (*P*6/*mmm*; *a* = 3.656(2) Å; *c* = 3.868(4) Å) (Supplementary Fig. 1). In the nanofibre syntheses, long structures with diameters ranging from 200 nm to 2 μm and lengths in excess of 10 μm were produced (Fig. 1). Atomic force microscopic (AFM) measurements confirmed these ranges of thicknesses in the different fibres observed (Supplementary Fig. 2). The morphology of the nanostructured material can be tailored by controlling the preparative conditions to yield different types of nanofibres (see “Methods”).

The growth of the one-dimensional (1D) nanostructures can be rationalised in terms of a self-assisted vapour–liquid–solid (VLS) mechanism. Under vacuum, Li_3_N will decompose at a temperature below the ambient pressure decomposition temperature (815 °C; *ΔH*_(298K)_ = −171.3 +/− 7.7 kJ mol^−1^)^7,8^ (Eq. 1), forming droplets of liquid Li, since the melting point of Li (*T* = 180.5 °C) lies well below the applied reaction temperatures. These Li seeds would act as nucleation sites for the N_2(g)_ species, which supersaturate the Li droplets and eventually lead to anisotropic growth and Li_3_N fibre formation. Energy-dispersive X-ray spectroscopy (EDX) data of the fibrous material did not show any traces of other metals such as Fe (from the wire used to support the reaction vessel), ruling out the possibility of Fe acting as a catalyst or seed for the formation of the fibres described here (Supplementary Fig. 3). Self-catalytic growth of GaN nanowires from Ga droplets and gallium/nitrogen vapour species has been observed previously^9^. The comparison to GaN is apposite in that both binary nitrides are formed from low melting point metals.

1 $$2{\mathrm{Li}}_3{\mathrm{N}}_{({\rm{s}})} \to {\mathrm{6Li}}_{{\mathrm{(l)}}} + {\mathrm{N}}_{2{\mathrm{(g)}}}$$

**Fig. 2:**
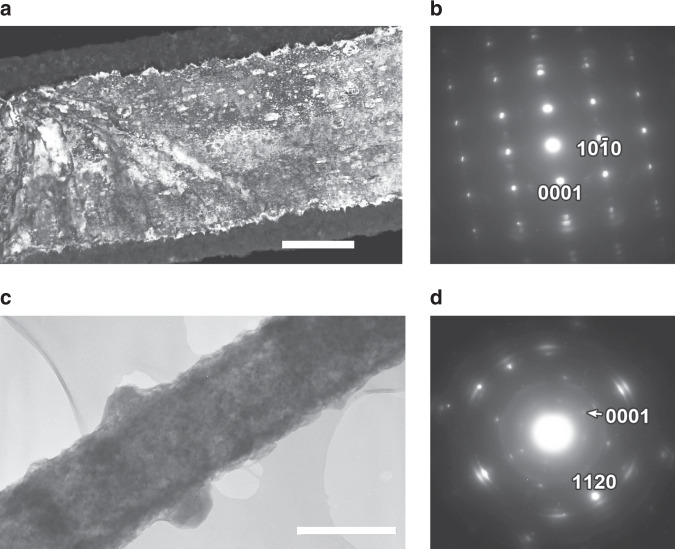
Transmission electron microscopic (TEM) images and selected area electron diffraction (SAED) patterns of type I and type II fibres. **a** High-magnification dark-field TEM image of a type I fibre (Li_3_N core and an LiOH outer layer) and **b** corresponding SAED pattern in the <$\overline 1$2$\overline 1$0> projection demonstrating the <10$\overline 1$0> nanofibre growth direction; **c** bright-field TEM image of a type II fibre and **d** corresponding SAED pattern in the <01$\overline 1$0> projection (with arcs due to an LiOH surface layer) showing the <0001> growth direction. Scale bars in **a** and **c** correspond to 500 nm.

Two types of single-crystalline straight fibres with different growth orientations (I and II) form under varying reaction conditions (see Supplementary Tables 1 and 2): type I nanofibres (Fig. 2a, b) exhibit a growth direction of <$10\bar 10$>, whereas, conversely, type II nanofibres exhibit a growth direction of <0001> (Fig. 2c, d). Typically, type II nanofibres were found together with a few fibres and nanosheets with zig-zag morphology (Supplementary Figs. 4 and 5). Thus Li_3_N nanofibres exist in which the hexagonal [Li_2_N] layers are stacked either parallel or perpendicular to the principal fibre axis. Carbon nanofibres can assemble in an analogous fashion, where graphene layers can be oriented either perpendicular or parallel to the principal fibre axis^10,11^. Our experimental evidence shows that the pressure within the reaction vessel is likely to be the crucial parameter for the preferential growth of type I and II nanofibres, i.e. under identical reaction times and temperature, type II fibres will be favoured over type I fibres at higher reaction pressures (Supplementary Tables 1 and 2). There are several examples in the literature that describe how the total pressure and/or precursor partial pressure can control the growth direction of fibres; these preferences can be both thermodynamically and kinetically driven^12^. Indeed, in this respect, our density functional theory (DFT) calculations suggest that type I nanofibres (−325.44 eV Li_3_N unit^−1^ see “Methods”) are more thermodynamically stable than the equivalent type II fibres (−324.59 eV Li_3_N unit^−1^). The dependence of growth direction on seed concentration in layered BN and GaN fibres has been previously reported^13,14^. Moreover, it has been well documented that nanowire growth direction is directly impacted by pressure across diverse systems of varying complexity (e.g. in semiconductors such as Si, In_2_O_3_ and InN)^15–18^. One might expect an increased Li seed concentration at elevated reaction pressure. An increase in N_2_ partial pressure (concentration) and the N:Li ratio at the growth interface should lead to the formation of Li-N planes and type II fibre propagation. Local variation in pressure and N:Li stoichiometry might, therefore, account for the formation of zig-zag (kinked) nanofibres and sheets observed in type II samples (Supplementary Figs. 4 and 5); analogous behaviour has been noted for Si and InN nanowires, for example^16,17^.

Raman spectra from nanostructured Li_3_N (fibres type I and type II) and bulk Li_3_N showed one prominent peak at ca. 580 cm^−1^, attributable to the only Raman active mode of Li_3_N, i.e. *E*_2g_ (Supplementary Fig. 6). This phonon mode corresponds to the displacement of the Li(2) and N atoms along the *ab* plane in Li_3_N^19^ and is consistent with group theory predictions and the spectra of bulk Li_3_N^20^. We found that the *E*_2g_ mode band was slightly blue-shifted (by ca. 5 cm^−1^) with respect to the bulk for both type I and type II fibres. Raman spectra of microcrystals of structurally similar graphite and BN^21,22^ and of BN nanotubes^23^ also revealed that the *E*_2g_ band broadens and shifts to a higher frequency as particle size decreases. Ab-initio calculations on BN single-walled nanotubes attribute the blue shift in the *E*_2g_ mode (of 5 cm^−1^) to a shortening of the sp^2^ bonds with respect to the bulk^23^. The additional bands observed at ca. 500 cm^−1^ and 650 cm^−1^ in both bulk and nanoscale Li_3_N samples were assigned to the acoustic (A) and optical phonon (O) modes 2 TA (z, K), 2 TO(A) and 2 LA(A) (T = transverse, L = longitudinal) by direct comparison with the second-order Raman spectra of Li_3_N^19,24^. Bands corresponding to the second-order phonon mode (2 LA (M, K)) were also observed in the 250–460 cm^−1^ region. The presence of these modes is attributed to the resonant conditions established when using laser irradiation, i.e. at 2.33 eV^25^.

**Fig. 3:**
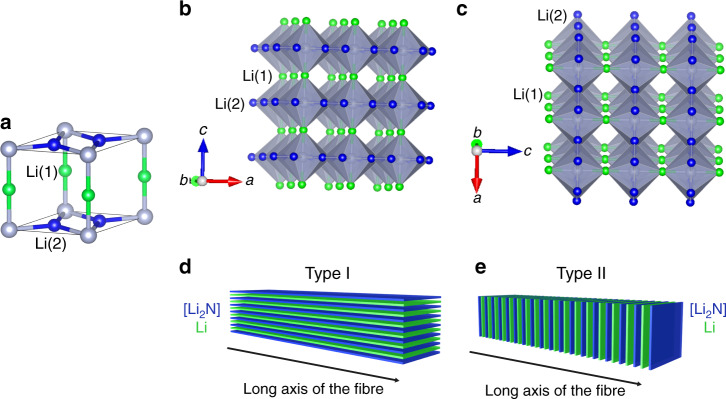
Schematic diagrams illustrating the Li_3_N crystal structure and type I and type II nanofibres. **a** Li_3_N unit cell, showing Li(1) atoms (green spheres), Li(2) atoms (blue spheres) and N (grey spheres); polyhedral representations of Li_3_N showing Li(1) atoms and Li(2) atoms: **b** aligned in the *ab* direction and **c** perpendicular to the *ab* direction; corresponding depictions of **d** type I and **e** type II Li_3_N nanofibres, based on the orientation of the Li (green) and [Li_2_N] layers (blue) with respect to the long axis of the fibre.

The layered crystal structure of Li_3_N (hexagonal *P*6/*mmm*) shares salient symmetry features with those of hexagonal graphite and BN. [Li_2_N] layers contain Li atoms in trigonal planar coordination to N, ostensibly by analogy to sp^2^ hybridised carbon in the graphene layers of graphite or to boron and nitrogen in BN (Fig. 3)^1^. Given the formation of anisotropic fibres (types I and II; Fig. 3) like layered chalcogenides, MX_2_ (M = early transition metal; X = S, Se), it may be tempting to classify Li_3_N as an extension of this latter class of materials^26^. Importantly, however, what distinguishes Li_3_N from the above examples is the absence of a van der Waals gap; although anisotropic, Li_3_N is connected in the third dimension by Li atoms (forming infinite [-Li-N-Li-] chains).

### Li^+^ and H^+^ transport properties

**Fig. 4:**
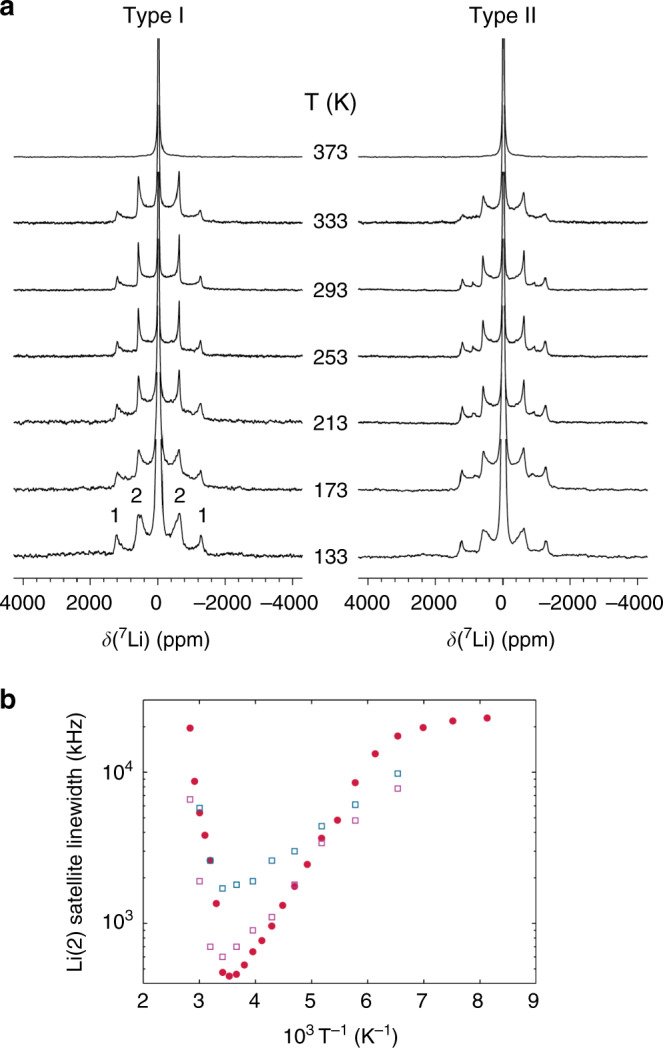
^7^Li NMR characterisation at variable temperatures of type I and II fibres. **a** Variable-temperature (VT) wideline ^7^Li NMR spectra recorded between 133 and 373 K for type I (left) and type II (right) Li_3_N nanofibres. 1 and 2 denote satellite bands for the two-coordinate Li(1) site and three-coordinate Li(2) site, respectively; **b** temperature variation of the linewidth of the Li(2) satellites for type I (magenta) and II (blue) Li_3_N nanofibres and bulk Li_3_N (red)^28^ extracted from the VT NMR by fitting a simulated powder lineshape.

Given the exceptional Li^+^ fast ionic conducting properties of bulk Li_3_N, we wished to understand how the ionic transport might vary as a consequence of nanostructuring. Variable-temperature wideline ^7^Li solid-state nuclear magnetic resonance (NMR) measurements between 133 and 373 K enabled the local structure of the type I and type II nanofibres to be probed and allowed the Li^+^ ion transport properties of the Li_3_N nanofibres to be determined (Fig. 4, Supplementary Note 2 and Supplementary Figs. 7 and 8). The line narrowing observed particularly for the Li(2) satellite lines between 133 and 293 K can be interpreted in terms of intra-layer Li^+^ diffusion, by analogy with the corresponding behaviour for bulk Li_3_N^27^. Similarly, the broadening that occurs >293 K and eventually causes both sets of satellites to disappear into the baseline results from inter-layer diffusion via an exchange of Li^+^ between the Li(1) and Li(2) sites. Assuming simple Arrhenius behaviour, the activation energy for the former intra-layer process can be measured from the temperature variation of the linewidth and was found to be 0.075 and 0.053 eV for type I and II Li_3_N nanofibres, respectively, which should be compared with the value of 0.121 eV, previously obtained for bulk Li_3_N^28^ (Supplementary Table 3). Hence, Li^+^ ion hopping becomes more facile within the Li-N planes as a result of nanofibre formation. Electrochemical impedance spectroscopic measurements were attempted on these samples to corroborate the conductivity results obtained by NMR. However, the high temperatures required to sinter pellets pressed from the Li_3_N nanofibres (i.e. to reduce grain boundary resistances) compromised their nanostructured morphology, so although high values of conductivity could be obtained (ca. 1 × 10^−3^ S cm^−1^), we could not treat the data obtained as representative of the fibres.

High Li^+^ ion conductivity is a fundamental requirement for solid electrolytes to compete against organic electrolytes in lithium-ion batteries. To date, only a few sulfide-type electrolytes have shown comparable ionic conductivities to commercial organic electrolytes^29^. Thus nanostructuring of solid electrolytes might be a plausible approach to increase ionic conductivity. This field is only at its infancy, with most of the work conducted on the Li_7_La_3_Zr_2_O_12_ garnet solid electrolyte (as a sintered ceramic or as an inorganic filler component in solid composite polymer electrolytes)^30^. Nevertheless, given the low decomposition potential of Li_3_N (ca. 0.45 V vs. Li^+^/Li)^31^ and the enhanced electronic conductivity expected in these nanostructured materials when compared to the bulk (see “DFT calculations” section), the use of Li_3_N as a solid electrolyte seems implausible from a practical point of view. On the other hand, in lithium-containing hydrogen storage materials, e.g. the lithium amide–lithium imide system, proton mobility has been intrinsically linked to Li^+^ ion diffusion^32^. Hence, we expected that the high Li^+^ ion mobility observed in these Li_3_N nanofibres may lead to improved hydrogen absorption properties.

Preliminary volumetric experiments to determine the hydrogen uptake properties of the nanostructured Li_3_N material were conducted by differential pressure analysis (DPA). As compared to bulk samples of highly crystalline, single-phase Li_3_N, the initial H_2_ uptake for the nano-Li_3_N material was found to be lower (8.9 vs. 10.5 wt%). The latter uptake is typical for pristine bulk material and matches the theoretical capacity from a two-step reaction mechanism (Eq. 2)^6^:

2 $${\mathrm{Li}}_{\mathrm{3}}{\mathrm{N}} + 2{\mathrm{H}}_{\mathrm{2}} \leftrightarrow {\mathrm{Li}}_{\mathrm{2}}{\mathrm{NH}} + {\mathrm{LiH}} + {\mathrm{H}}_2 \leftrightarrow {\mathrm{LiNH}}_2 + 2{\mathrm{LiH}}.$$

One might postulate that the reduction in initial gravimetric capacity in the nanofibres is a result of surface hydrolysis (passivation) on handling in air (as observed from transmission electron microscopy (TEM)/selected area electron diffraction experiments; Supplementary Note 1 and Supplementary Fig. 9). Nevertheless, there is a greater than threefold increase in the rate of hydrogen sorption for nanocrystalline Li_3_N with respect to the bulk material in the first cycle, which dramatically improves further in subsequent uptake cycles (Supplementary Fig. 10). The microstructure of the dehydrogenated nitride is retained through cycling (from imide to amide and vice versa) and hence the (de)hydrogenation process is pseudomorphic and reversible (Supplementary Fig. 11). The enhanced hydrogen uptake behaviour of the nanofibres is broadly analogous to the uptake kinetics observed when Li_3_N is impregnated in mesoporous carbon or used in carbon nanocomposites (also at a reduced sorption temperature of 200 °C and which exhibit a hydrogen desorption enthalpy that is half that of the bulk material)^33^. Unlike these composites, however, the nanofibres do not suffer from the gravimetric capacity penalty imposed by an inactive component. Computational studies have demonstrated that pseudo-molecular (Li_3_N)_*n*_ (*n* = 1–7) clusters would bind H_2_ via coordinatively unsaturated Li atoms with an adsorption energy approximately an order of magnitude smaller than that of the bulk material^34^. By extension, earlier DFT calculations show that the most favourable adsorption sites for both H_2_ and dissociated H atoms are to N positions on the (001) surface of Li_3_N^35^. Semi-quantitatively at least, both prior studies would indicate that fabrication of narrow nanofibres or thin nanosheets of Li_3_N should produce storage materials with improved sorption kinetics. Thus the design and optimisation of anisotropic Li-N(-H) nanomaterials could prove a useful strategy towards attaining a storage solution that meets the challenging criteria required for implementing hydrogen as a fuel for transport. Further studies should establish whether this is indeed the case.

### Calculations on nanostructured α-Li_3_N electronic properties

**Fig. 5:**
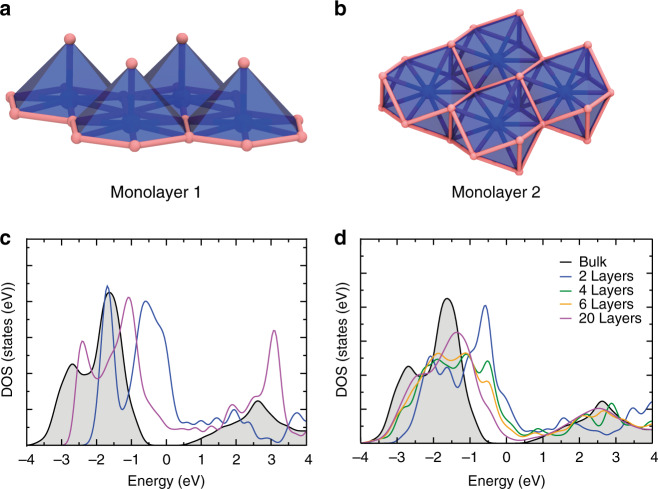
Li_3_N monolayer configurations and electronic structures determined from density functional theory calculations. Calculated Li_3_N monolayer structures of: **a** form 1; **b** form 2; **c** comparison of the total DOS for bulk Li_3_N (black) with form 1 (blue) and 2 (magenta) monolayers, respectively; **d** evolution of the total DOS for increasing numbers of Li_3_N layers (2 (blue), 4 (green), 6 (yellow) and 20 (magenta)) as compared to that of the bulk material (black).

Electronic properties were computed on α-Li_3_N using DFT to compare the behaviour of a monolayer of Li_3_N with respect to the bulk material and to develop an understanding of the evolution of electronic structure with the number of layers as compared to graphene and other van der Waals nanostructures (vdWNs). Our calculations confirm that bulk α-Li_3_N is a semiconductor with an indirect bandgap of *E*_g_ = 1.3 eV, in agreement with several previous first-principles calculations of the α-Li_3_N electronic properties^36–39^. The electronic bandgap predicted by DFT is notably smaller than the experimental optical bandgap of 2.18 eV for Li_3_N single crystals^40^. This discrepancy is due to the well-known DFT problem in underestimating conduction band state energies^41,42^. Unlike layers in vdWNs, a single [Li_2_N] layer is unstable and computational optimisation produces two monolayer structures from the combination of [Li_2_N] and [Li] planes, forms 1 and 2 (Fig. 5a, b), of which the latter is more stable by −0.574 eV.

Although the profile of the single-layer density of states (DOS) more closely resembles that of the bulk material for the form 2 monolayer, both single-layer forms are predicted to be metallic (Fig. 5c). In each case, the DOS below and approaching the Fermi level is attributable to N states, whereas the higher states of the conduction band are composed predominantly of Li states. As the number of layers in a Li_3_N slab is increased, the total DOS at the Fermi level decreases and a clear tendency towards an opening of a bandgap is observed (Fig. 5d). Li_3_N nanosheets composed of up to 20 layers (75.6 Å thick), for example, remain metallic, although using accurate quantum chemical approaches it is difficult to predict a critical thickness at which the metal–semiconductor transition occurs. The evolution of the electronic structure contrasts markedly with graphene, where there is a relatively abrupt change from zero bandgap semiconducting to semi-metallic graphite-like behaviour as the number of stacked monolayers increases^43^. Experimental data suggest that the bandgap in BN 5-layer nanosheets (5.56 eV) is smaller than that calculated for a BN monolayer (6 eV), but both values are larger than that of the bulk material (5.2–5.4 eV) and the nitride remains an insulator^44–46^.

## Discussion

In summary, anisotropic 1D Li_3_N nanostructured materials can be formed by broad analogy to sp^2^-like layered solids such as graphite and boron nitride, despite the absence of a van der Waals gap in Li_3_N. Two distinct types of Li_3_N fibres have been identified, here denoted as type I and type II, which differ in the orientation of the [Li_2_N] layers (parallel or perpendicular) with respect to the long axis of the fibre. The formation of 1D structural variants of Li_3_N has important consequences for the transport of Li^+^ (and H^+^) ions, which lead to demonstrable differences in diffusion behaviour compared to the bulk material. DFT calculations have shown that the electronic properties of these Li_3_N materials appear scalable with size and shape.

Given their predicted metallic-like behaviour and high Li^+^ ion mobility, we anticipate that these nano-based Li_3_N materials could play an important role as additives in high-power Li-based batteries, which demand rapid ionic and electronic kinetics. Currently, bulk and surface-passivated Li_3_N has been used as a pre-lithiation additive in various cathode materials (e.g. LCO, NMC)^47,48^ to offset the first-cycle lithium loss and thus to improve the overall energy density of the material. Furthermore, bulk Li_3_N has been used as a protective coating for Li metal anodes in Li-ion and Li-S batteries to avoid dendrite growth and parasitic reactions between polysulfides and Li metal^49,50^. Importantly, the nanostructured materials allow for the rapid growth of an insulating surface passivation layer (e.g. formed from Li_2_CO_3_) conferring higher chemical stability that could eliminate reactions at the electrolyte interface. From a different perspective, the superior Li^+^ ion diffusion in the Li_3_N nanofibers leads to improved hydrogen absorption/desorption properties in the Li–N–H system given the intrinsic relationship between proton and lithium-ion conduction in this system. Thus, by analogy, it may be conceivable to revisit other materials related to the Li–N–H family of compounds to explore the impact of nanoscaling on hydrogen storage and generation more widely.

Our studies clearly demonstrate that combining an s-block element with nitrogen under the appropriate synthetic conditions can lead to the formation of anisotropic nanomaterials. Nanostructuring has a palpable influence on many chemical and physical properties in the Li–N system and there are likely to be other exciting phenomena and behaviours to be discovered. We expect that this work will stimulate further research on this system and those containing other s-block elements.

## Methods

### Synthesis of type I Li_3_N nanofibres

α-Li_3_N (ca. 0.1 g) prepared from the nitridation of a sodium/lithium alloy^28^ was contained within an iron foil liner that was placed inside a stainless-steel crucible. The crucible was suspended by an iron wire inside a long, 3 cm internal diameter stainless-steel reaction vessel that was water-cooled at the upper end. The vessel was evacuated to a pressure of ca. 5 Pa (a range of 4–6.7 Pa), then sealed and heated to 1023–1073 K in a vertical furnace for 6 days. Finally, the furnace was cooled to room temperature. Supplementary Table 1 provides detailed reaction conditions for each of the syntheses performed that yielded type I Li_3_N nanofibres.

### Synthesis of type II Li_3_N nanofibres

α-Li_3_N (ca. 0.1 g) prepared from the nitridation of a sodium/lithium alloy^28^ was contained within an iron foil liner that was placed inside a stainless-steel crucible. The crucible was suspended by an iron wire inside a long, 1.5 cm internal diameter stainless steel reaction vessel that was water-cooled at the upper end. The vessel was evacuated to a pressure of ca. 10 Pa (a range of 9.3–10.7 Pa), then sealed and heated to 1023–1043 K in a vertical furnace for 6 days. Finally, the furnace was cooled to room temperature. Supplementary Table 2 shows detailed reaction conditions for each of the syntheses performed that yielded type II Li_3_N nanofibres.

The handling of the reactants and products took place in a nitrogen-filled glovebox (Saffron Scientific Ltd.; O_2_ < 5 ppm; H_2_O < 10 ppm) during the syntheses of both types of nanofibres. In both cases, on cooling, red/dark purple fibrous material (10–40 mg) was found deposited on the Fe wire above the reaction crucible. EDX spectra of all nanomaterials reveal peaks only from nitrogen (and oxygen from brief air exposure). Given that Li is not detectable by EDX and that no peaks from lithium metal (or other Li-containing phases) are observed in solid-state NMR spectra, the results are consistent with the presence of single-phase Li_3_N. The absence of metal impurity peaks corroborates a self-assisted growth mechanism.

### Powder X-ray diffraction

PXD data were collected using a Philips X’Pert *θ*–2*θ* diffractometer with a PW3710 control unit using Cu Kα radiation (*λ* = 1.5418 Å), operating at 40 kV and 40 mA. Samples were run within a bespoke air-tight aluminium holder with Mylar windows^51^. Data were collected in the range from 5 to 80° 2*θ* in steps of 0.025° s^−1^. Sample preparation for analysis took place in an N_2_-filled glovebox. Lattice parameters were refined by least-squares fitting using the CELL software package^52^.

### Scanning electron microscopy (SEM)

A Philips XL30 ESEM-FEG instrument was used for SEM and EDX characterisation. Experiments were performed in high vacuum mode with an applied accelerating voltage of 15 kV. Samples were loaded onto aluminium stubs using adhesive carbon tabs and the transfer was performed under a stream of flowing N_2_ gas.

### Atomic force microscopy

AFM sample preparation consisted of dispersing the samples in n-propyl acetate in an Ar-filled glove box. Then a droplet of the concentrated Li_3_N solution was placed onto a silicon wafer for measurement. AFM analyses were carried out using a Keysight 5500 instrument in tapping mode to produce topographical information of the samples.

### Transmission electron microscopy

TEM analyses were performed using different instruments under varying operating conditions. Measurements performed at 80 kV were conducted using a JEOL JEM-2200MCO FEGTE. Samples were loaded in an N_2_-filled glovebox onto a custom-made air-tight holder to minimise air exposure during transfer. Analyses performed at 200 kV were made using either a JEOL JEM-2000FX II TEM or an FEI Tecnai G^2^ 20 TEM. TEM samples were prepared by depositing the Li_3_N dry onto a 3-mm holey carbon film copper grid in an N_2_-filled glovebox. Each grid was placed within a sealed container and transferred to the instrument under a stream of N_2_. In both cases, a small condenser aperture was used to reduce beam damage (the result of which is evident in Supplementary Fig. 12) and evaporation due to the instability of nanoscale Li_3_N under the beam.

### ^7^Li NMR spectroscopy

Wideline ^7^Li NMR spectra of Li_3_N nanomaterials were recorded from 133 to 453 K at a Larmor frequency of 116.6 MHz on a Varian InfinityPlus Spectrometer equipped with a single-resonance broadband probe. Spectra were obtained using an EXORCYCLED solid-echo sequence with *π*/2 pulses of 1.3 µs and an echo delay of 16.0 µs. Relaxation delays between 0.5 and 10 s were used depending on the temperature. Chemical shifts are referenced externally to aqueous LiCl.

### Raman spectroscopy

Raman spectra were collected using a Horiba-Yvon LabRam HR spectrometer with a confocal microscope at room temperature using a 532-nm laser with a 600 g mm^−1^ grating and a Synapse CCD detector. Samples were mounted in sealed glass capillaries in an N_2_-filled glovebox to avoid air exposure. A ten times reduced incident laser power (15 mW) was used together with a 100-nm aperture to avoid damaging the material under the beam during analysis.

### Hydrogen storage measurements

Volumetric hydrogen uptake measurements were performed by loading ca. 0.2 g sample into a Swagelock tube in an N_2_-filled glovebox and sealed using parafilm. The tube was connected to the DPA apparatus^53^ and the parafilm was seal-broken using a flow of helium gas. The sample was evacuated before exposure to hydrogen and heated to the reaction temperature. H_2(g)_ and He_(g)_ were dried using a liquid nitrogen trap before use. Hydrogen uptake was performed at 10 bar at 200 °C. After reaching equilibrium, the sample was desorbed at 200 °C before performing the next absorption cycle.

### DFT calculations

Electronic properties of bulk and nano-sheets of α-Li_3_N were calculated at the gradient-corrected DFT level by applying the Perdew–Burke–Ernzerhof^54^ exchange-correlation functional as implemented in the SIESTA program package^55^. Electronic states were expanded by a double-ζ plus polarisation basis set with norm-conserving Troullier–Martins pseudopotentials^56^ for the description of core levels and a plane-wave representation of the charge density with a cut-off of 240 Ry. The reciprocal space was sampled by a Monkhorst–Pack grid^57^ of 16 × 16 × 32 *k*-points in the Brillouin zone (16 × 16 × 1 *k*-points in the case of nanosheets). Models of nanosheets are composed of an increasing number of unit cell replicas constructed in the direction perpendicular to the nano-sheet plane and a 15-Å-thick vacuum region. Geometries of all systems were relaxed until a maximum gradient of 0.02 eV Å^−1^ on forces was reached. In the calculations of the total energies of type I and type II nanofibres, the model structures were built with an increasing number of replicas of the unit cell in the non-periodic directions to achieve the convergence in terms of energy per number of replicas (14 replicas were used for type I nanofibre and 8 × 8 for type II nanofibre). An MP *k*-points sampling with 16 *k*-points in the periodic directions has been applied (two directions for type I nanofibre and one direction for type II nanofibre).

## Supplementary information

Supplementary Information

## Data Availability

The authors declare that the data supporting the findings of this study are available within the paper and its supplementary information files.
