# Supplementary material for: Low dimensional nanostructures of fast ion conducting lithium nitride
Source: Nat Commun. 2020 Sep 8;11:4492. doi: 10.1038/s41467-020-17951-6 (PMC7479578; doi:10.1038/s41467-020-17951-6)
Supplement: Supplementary file 1 — Supplementary Information [file 41467_2020_17951_MOESM1_ESM.pdf]

Supplementary Information

Low dimensional nanostructures of fast ion conducting lithium nitride

Tapia-Ruiz et al.

Table of contents

Supplementary Figures………………………………………………………………………………3

Supplementary Tables……………………………………………………………………...15

Supplementary Notes……………………………………………………………………....18

Supplementary References………………………………………………………………..20

List of Supplementary Figures

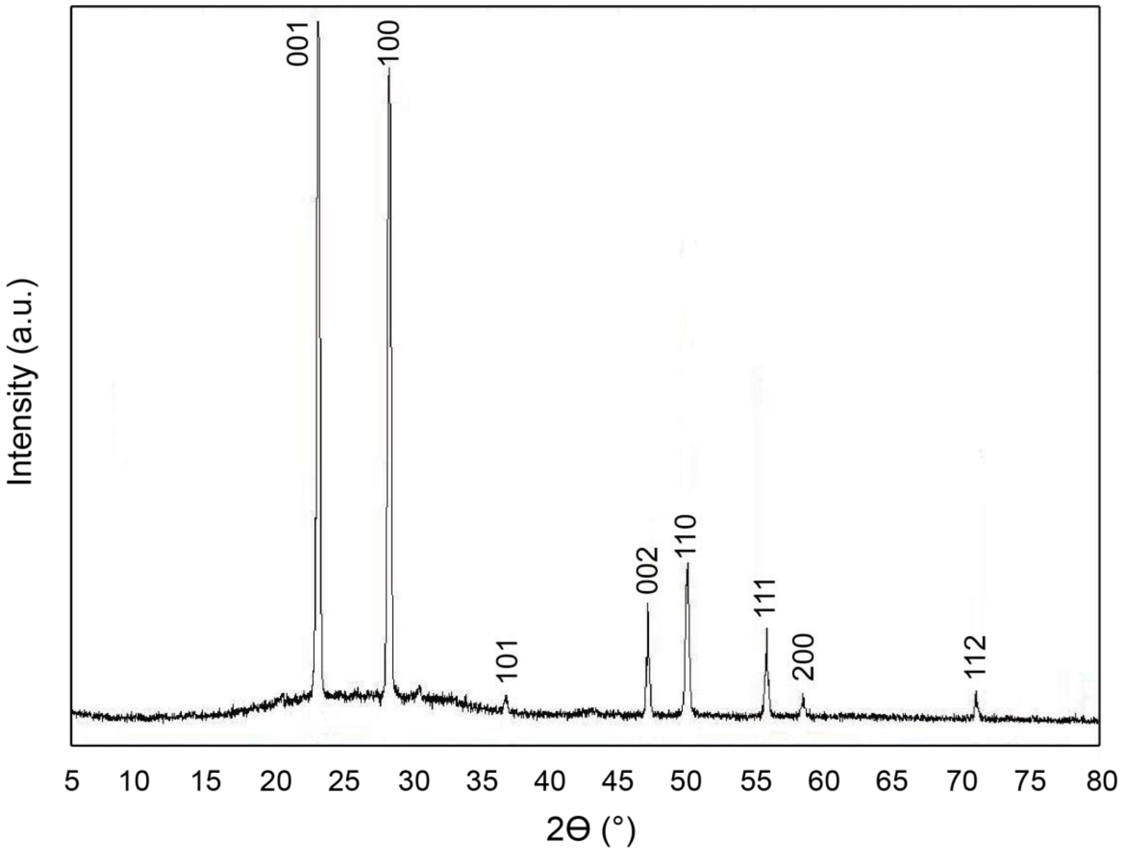

Supplementary Figure 1. Powder X-ray diffraction pattern for nanostructured Li_3N. Bragg reflections were indexed using the hexagonal space group *P*6/*mmm* (*a* = 3.656(2) Å and *c* = 3.868(4) Å). Miller indices of the respective α-Li_3N Bragg peaks are shown.

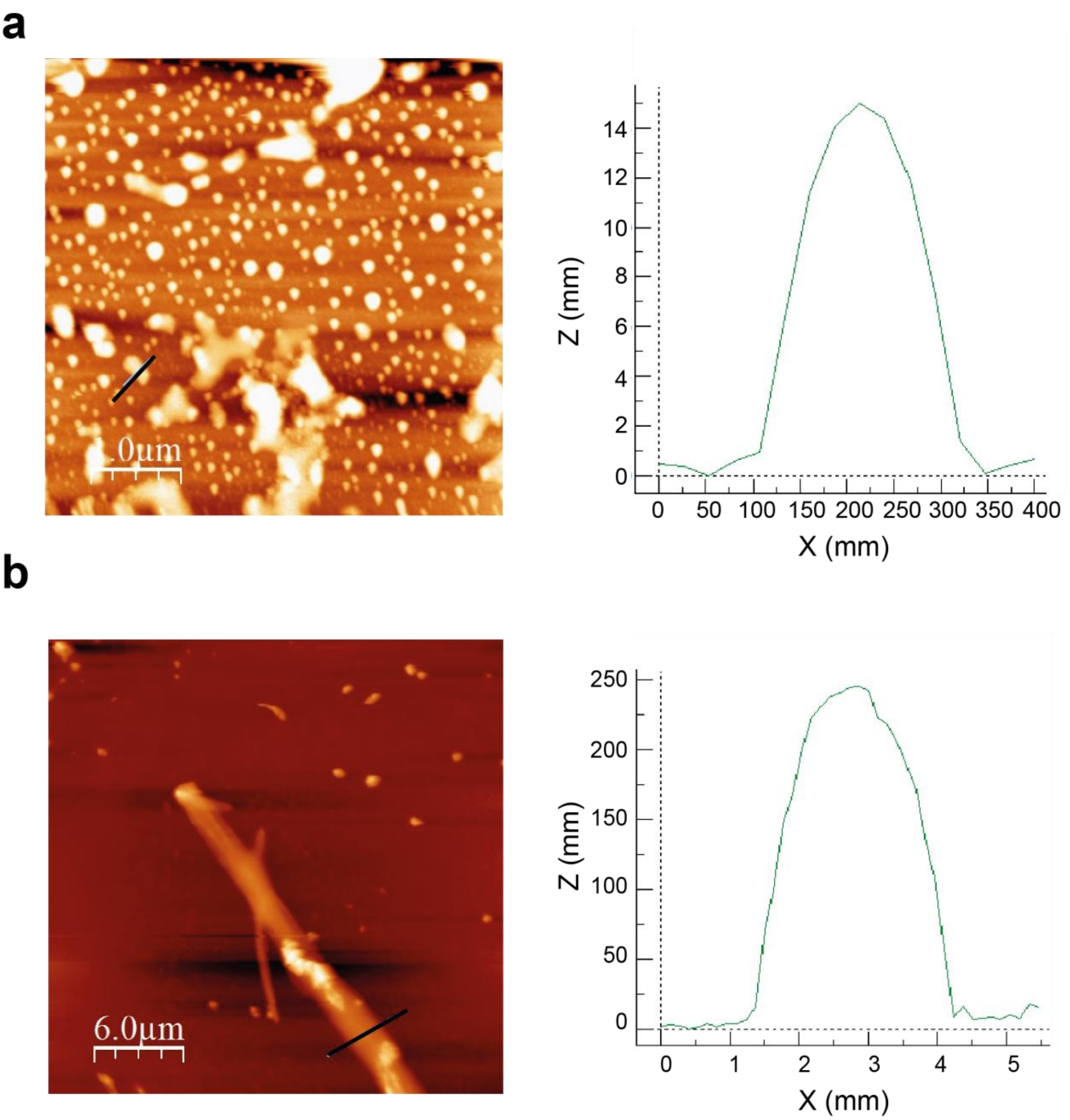

Supplementary Figure 2. AFM images (tapping mode) and spatial profiles of two distinct types (a and b) of nanostructured Li_3N specimens (transferred into the instrument suspended in N-Propyl acetate).

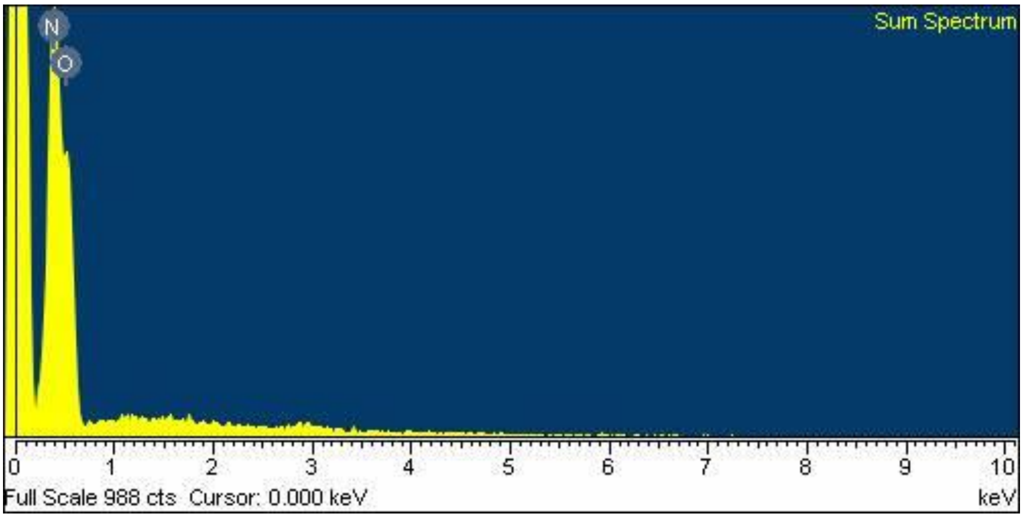

Supplementary Figure 3. EDX spectrum of Li_3N nanofibres showing peaks corresponding to N (from Li_3N) and O (from LiOH). The spectrum confirms the absence of other elements in the fibres.

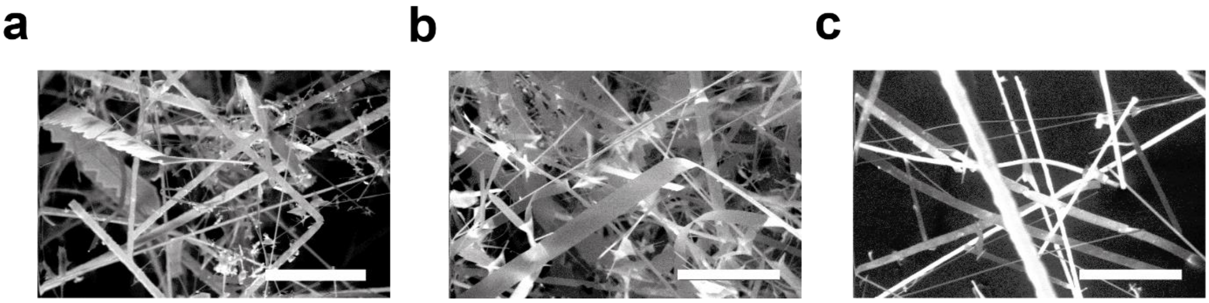

Supplementary Figure 4. SEM images of type II Li_3N nanofibres at different magnifications. The scale bars correspond to 100 μm in a) and to 50 μm in b) and c).

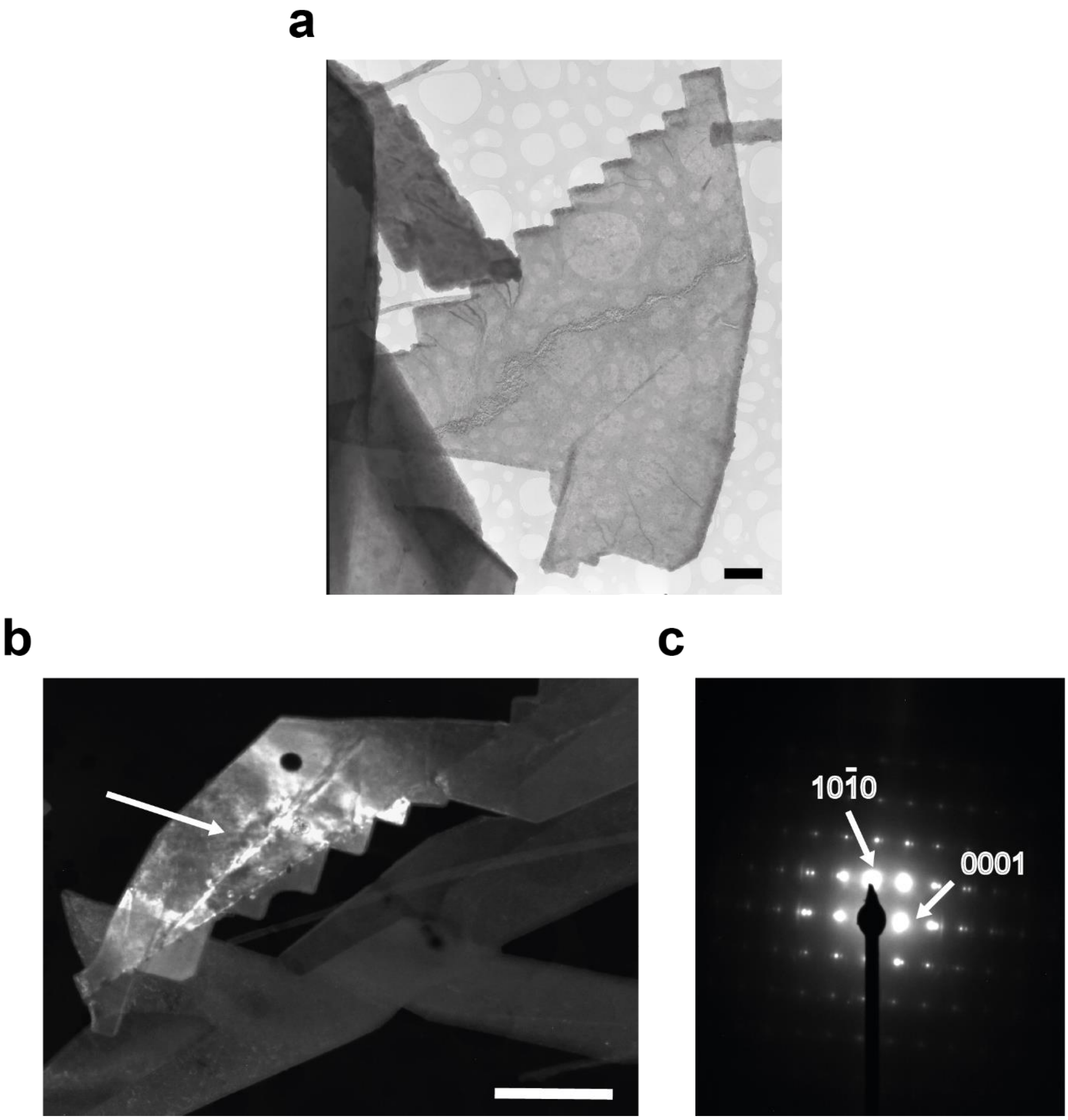

Supplementary Figure 5. a) Bright field TEM image of a crumpled Li_3N nanosheet. The scale bar corresponds to 1 μm; b) Dark field image of a nanosheet. The scale bar corresponds to 2 μm; and c) Selected area electron diffraction (SAED) pattern from the sheet in b) (taken at the point of the white arrow), demonstrating a $(1\bar{2}10)$ habit plane for the sheet, with sawtooth edges perpendicular to the $[10\bar{1}0]$ and [0001] directions.

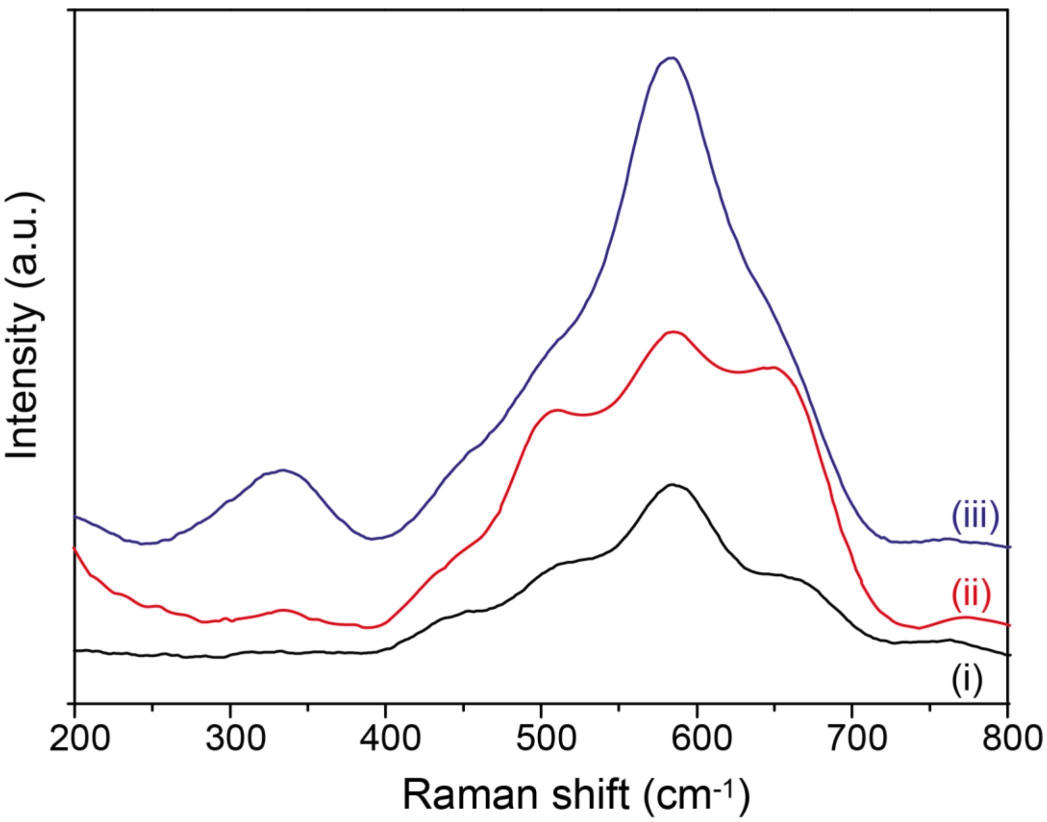

Supplementary Figure 6. Room temperature Raman spectra of (i) Li_3N type I nanofibres; (ii) type II nanofibres and (iii) bulk Li_3N.

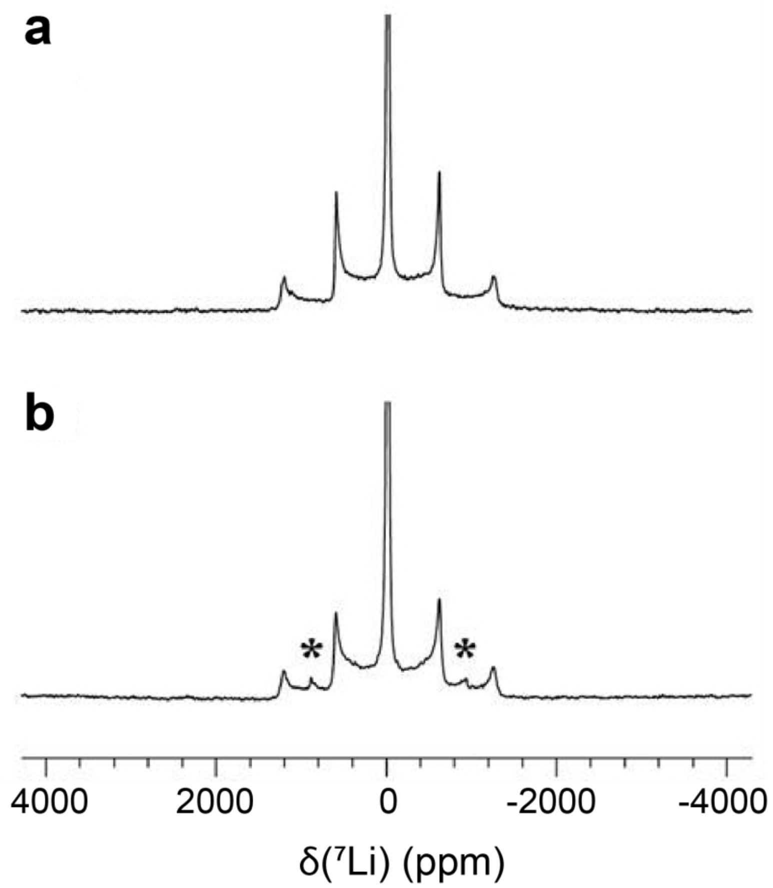

Supplementary Figure 7. Wideline ^{7}Li NMR spectra at 298 K for a) type I and b) type II nanofibres. An additional pair of satellites (indicated with asterisks) is observed for the type II nanofibers.

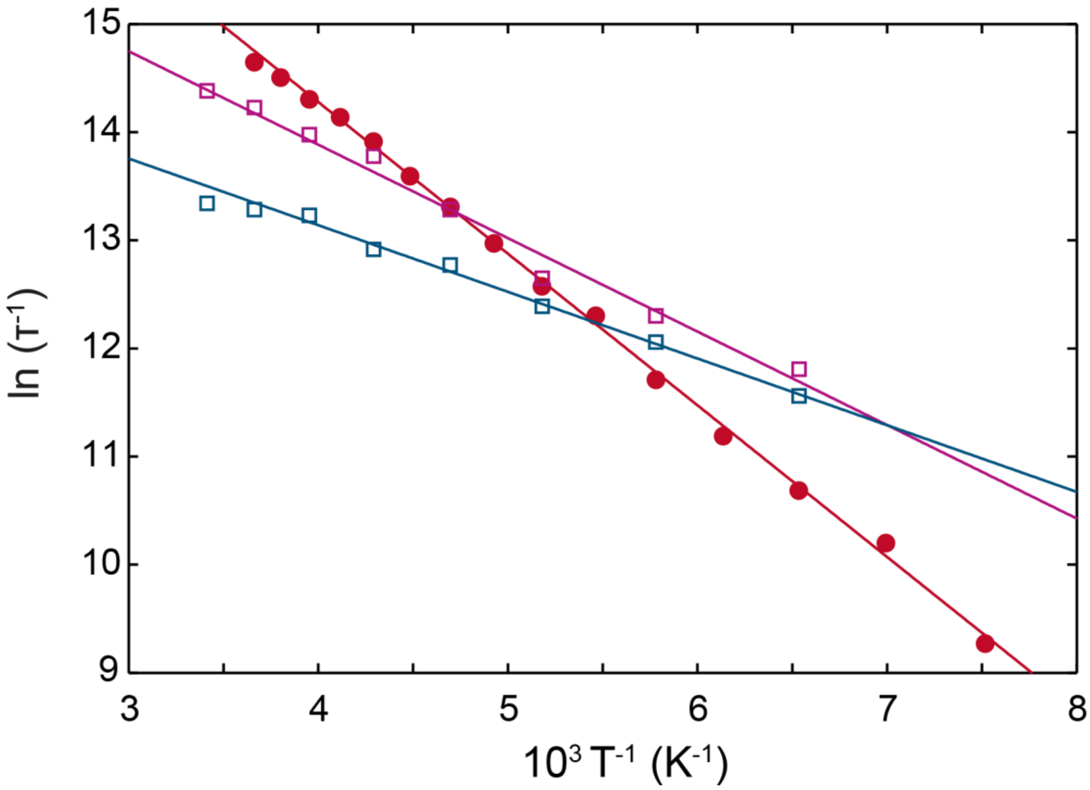

Supplementary Figure 8. Arrhenius plot of ln(τ_{NMR}^{-1}) against reciprocal temperature for intra-layer Li^+ diffusion for type I (magenta) and type II (blue) Li_3N nanofibres as compared to bulk Li_3N (red)[1]. All data points were extracted from the linewidth measurements shown in Figure 4b in the main paper.

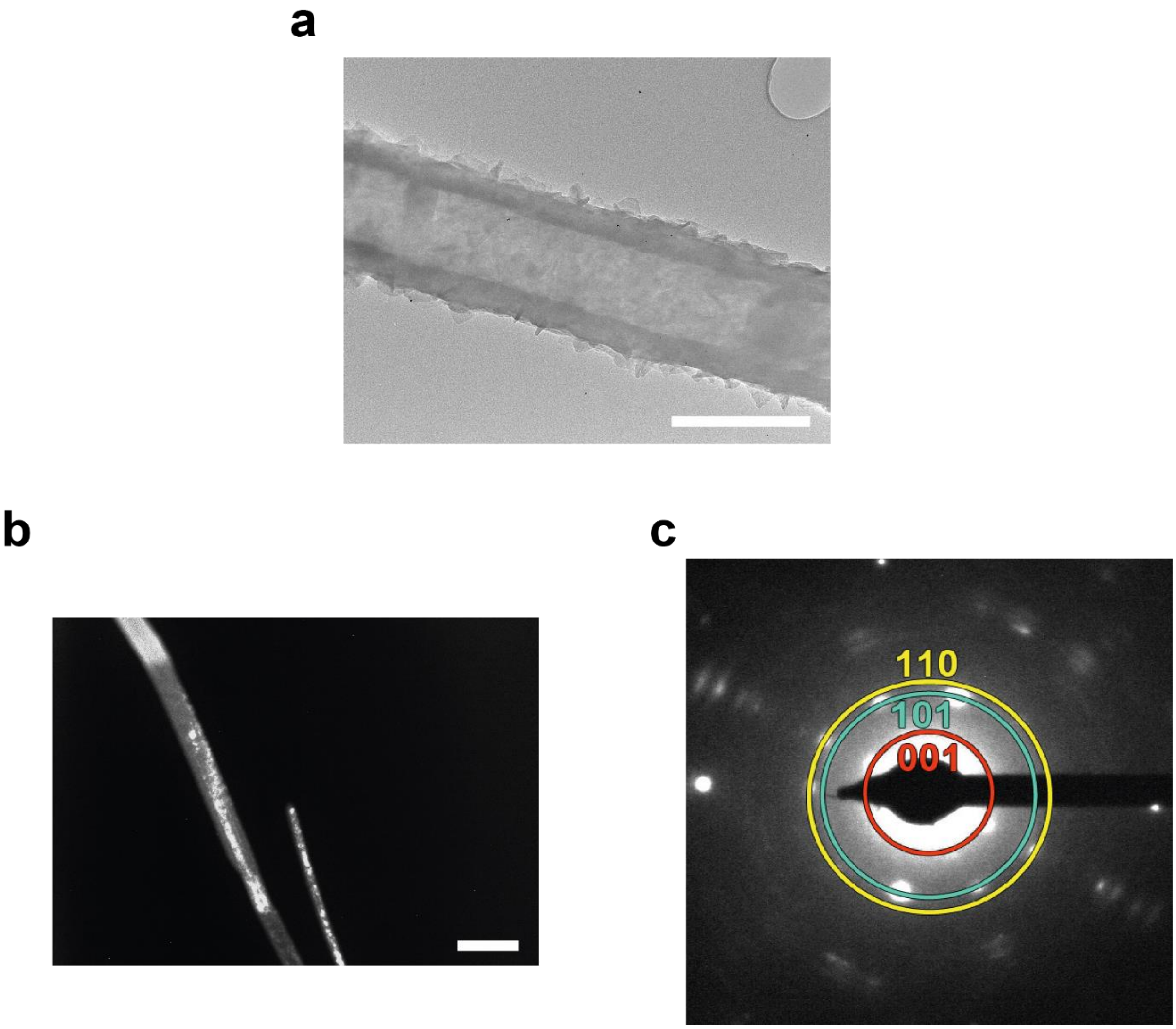

Supplementary Figure 9. a) Bright field high-resolution TEM image (80 kV) showing LiOH passivation layers on the surface of the Li_3N nanofibre. The scale bar corresponds to 500 nm; b) Dark field TEM image of a Li_3N fibre which has been almost completely converted to LiOH. The scale bar corresponds to 1 μm; and c) SAED pattern from the fibre in b). The three principal diffraction rings for LiOH are indicated.

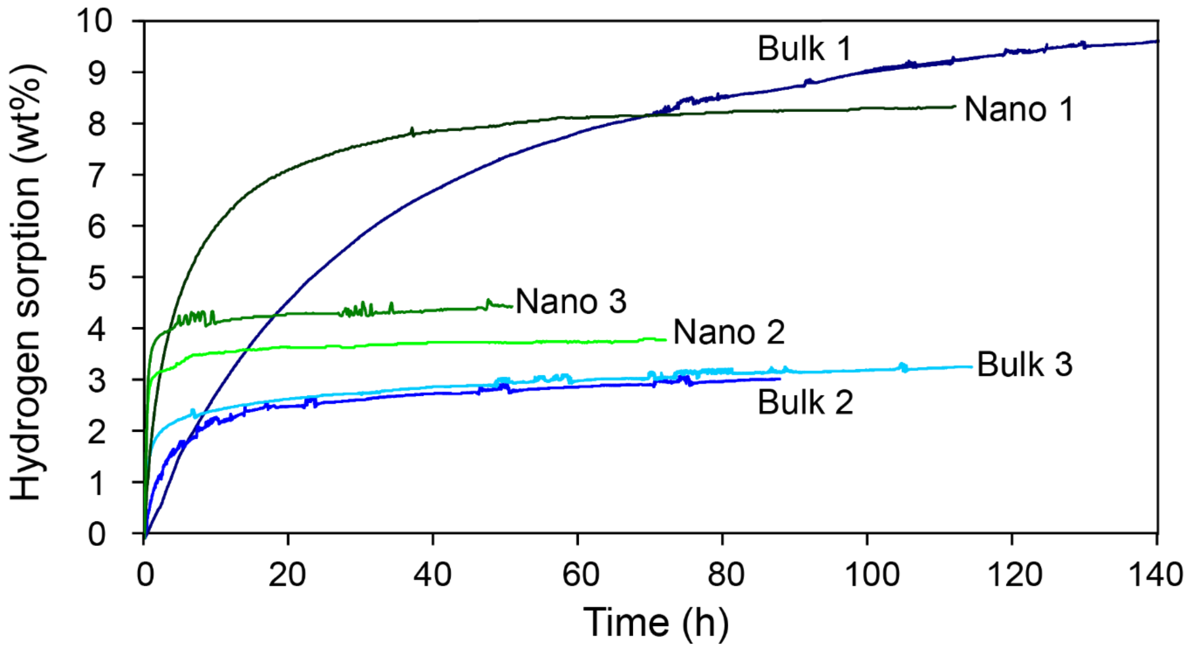

Supplementary Figure 10. Hydrogen uptake of nanostructured (green) and bulk (blue) Li_3N as a function of time, measured using a differential pressure volumetric method at 200 °C under 10 bar of hydrogen pressure. Dehydrogenation was performed at 200 °C. Bulk and Nano refer to crystalline bulk samples and nanofibre Li_3N samples respectively. Number designations refer to the respective uptake cycles (1-3). Total uptakes were: Bulk 1 - 10.5 wt.%, bulk 2 - 3.2 wt.%, bulk 3 - 3.5 wt.%; nano 1 8.9 wt.%, nano 2 4.4 wt.%, nano 3 - 5.3 wt.%.

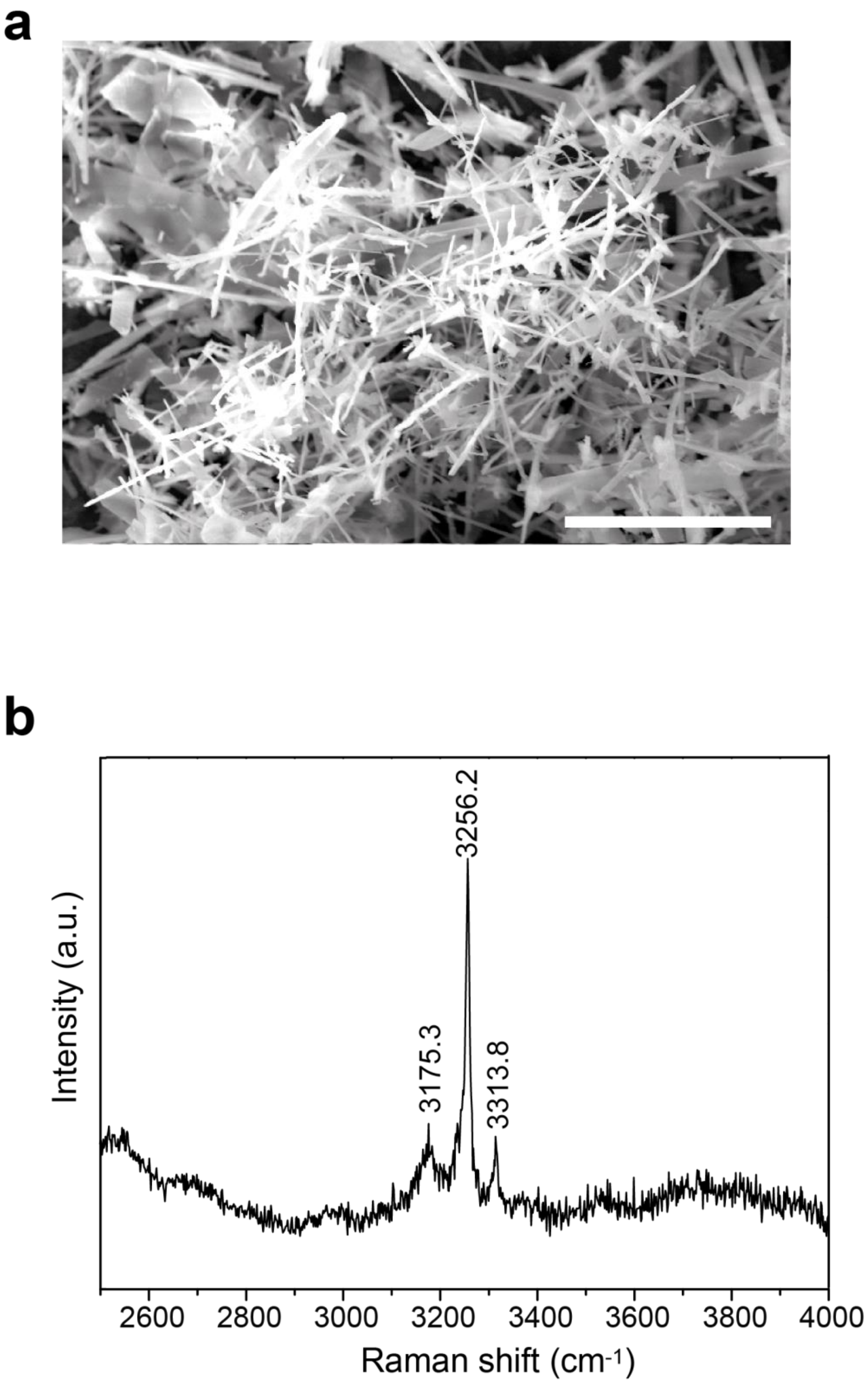

Supplementary Figure 11. a) SEM image of the nanostructured material after the first hydrogen desorption at 200° C, confirming that the morphology is maintained after cycling. The scale bar corresponds to 20 μm; b) Raman spectra (2500-4000 cm^{-1}) of the dehydrogenated product after one sorption (uptake-release) cycle showing a band at 3175.3 cm^{-1} that corresponds to the v(N-H_{sym}) vibration of Li_2NH; the band at 3313.8 cm^{-1} corresponds to the v(N-H_{asym}) mode of $LiNH_2$; and the band at 3256.2 cm^{-1} can be assigned to the v(N-H_{asym}) and v(N-H_{sym}) modes of Li_2NH and $LiNH_2$, respectively[2,3].

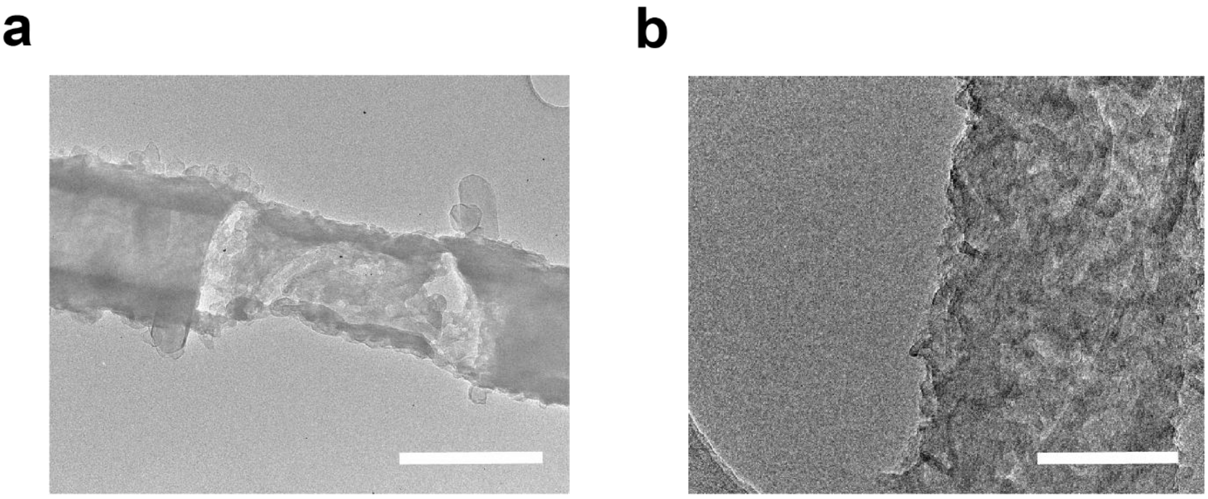

Supplementary Figure 12. a) High-resolution TEM image (80 kV) showing beam damage to a Li_3N nanofibre after beam exposure. Images a) and b) show evidence of thin nanoplates that appear to fragment from the fibre on extended beam exposure. The scale bars in a) and b) correspond to 500 nm and 100 nm, respectively.

List of Supplementary Tables

Supplementary Table 1. List of experiments with corresponding reaction parameters for the synthesis of type I fibres.

Expt. No.	*Initial pressure* / Pa	*T* / K	*Mass of Li_3N* / g	*Time* / days	*Product mass* / g	*Estimated Li_3N partial pressure* / KPa	*Reaction pressure*[a] / kPa
1	5.33	1023	0.08	6	0.015	126.98	126.99
2	5.33	1023	0.043	6	0.024	68.25	68.26
3	5.33	1073	0.081	6	0.021	134.86	134.86
4	4.00	1023	0.093	6	0.009	147.62	147.62
5	5.33	1023	0.082	6	0.007	130.16	130.16
6	5.33	1023	0.07	6	0.004	111.11	111.12
7	5.33	1023	0.072	6	0.009	114.29	114.29
8	6.66	1023	0.156	6	0.013	247.62	247.63
9	5.33	1023	0.113	6	0.035	179.37	179.37
10	5.33	1023	0.065	6	0.03	103.18	103.18

[a] The reaction pressure is the sum of the *Initial pressure* (column two) and the *Estimated Li_3N partial pressure* (column seven). The latter has been estimated using the ideal gas equation ($pV = nRT$), where T= furnace temperature, V= volume of the reaction vessel, n= number of moles of Li_3N, R= ideal gas constant.

Supplementary Table 2. List of experiments with corresponding reaction parameters for the synthesis of type II fibres.

Expt. No.	*Initial pressure* / Pa	*T* / K	*Mass of Li_3N* / g	*Time* / days	*Product mass* / g	*Estimated Li_3N partial pressure* / KPa	*Reaction pressure*[a] / kPa
1	9.33	1043	0.16	6	0.019	602.46	602.47
2	10.66	1023	0.228	6	0.016	842.04	842.06
3	10.66	1023	0.164	6	0.014	605.68	605.69
4	9.33	1043	0.118	6	0.038	444.31	444.32
5	10.66	1043	0.097	6	0.02	365.24	365.25

[a] The reaction pressure is the sum of the *Initial pressure* (column two) and the *Estimated Li_3N partial pressure* (column seven). The latter has been estimated using the ideal gas equation ($pV= nRT$), where T= furnace temperature, V= volume of the reaction vessel, n= number of moles of Li_3N, R= ideal gas constant.

Supplementary Table 3. Values of E_a, τ_0 and D (298K) for Li^+ intra-layer diffusion.

	E_a / eV	τ_0 / s	D (298 K) / $m^2 s^{-1}$
Bulk	0.121 ± 0.005	2.3 ± 0.7 x 10^{-9}	4.4 x 10^{-14}
Type I fibres	0.075 ± 0.008	1.7 ± 0.8 x 10^{-8}	2.1 x 10^{-14}
Type II fibres	0.053 ± 0.006	1.6 ± 0.8 x 10^{-7}	8.4 x10^{-15}

List of Supplementary Notes

Supplementary Note 1. SEM and TEM of hydrolysed Li_3N nanofibres

Li_3N nanostructures are extremely reactive in air and begin to hydrolyse after seconds of atmospheric exposure. Nanofibres exhibited layers of polycrystalline material at the surface (typically 10-50 nm thick) which were identified by SAED as LiOH from hydrolysis during the brief transfer to the microscope. (No equivalent hydroxide was detected by either PXRD or NMR). Prolonged air-exposure (of the order of minutes) resulted in the complete pseudomorphic transformation of the Li_3N structures to crystalline nanofibres of LiOH. Conversion of Li_3N to LiOH commences from the outside of the wire inward. This produces a tubular beam-stable outer shell of LiOH containing a Li_3N core (Supplementary Fig. 9). Analysis of a series of SAED patterns recorded across the width of a wire confirms this. Initially, SAED of the centre of a fibre gives a single crystalline Li_3N pattern of <111> projection. As the SAED aperture is traversed across the body of the wire, polycrystalline rings corresponding to LiOH form as diffraction arcs (Supplementary Fig. 9). The position of LiOH arc intensity is related to the orientation of the fibre and appears to correspond to the positions of certain Li_3N spots within the diffraction patterns. The relatively smooth Li_3N surface of the fibres is converted to a rougher, undulating texture in LiOH fibres and leads to the formation of curved joints between nanofibres.

Supplementary Note 2. ^{7}Li solid-state NMR

Wideline ^{7}Li (I=3/2) solid-state NMR spectra at 298 K for type I fibres (Supplementary Fig. 7a) show two sets of powder-broadened satellites with quadrupolar couplings (C_q) of 580 and 284 kHz respectively. These can be assigned to the Li(1) (inter-layer) and Li(2) (intra-layer) sites in the Li_3N fibre by direct comparison with the bulk material, for which the C_q values are essentially identical[4]. In contrast, for type II fibres (Supplementary Fig. 7b) an additional satellite with C_q = 424 kHz (labelled with asterisks in the figure) is observed. We have assigned this satellite to a new lithium environment in the fibres and one interpretation could be that this exists at the edge of the [Li_2N] layers and is coordinatively unsaturated (3-δ coordination number) at the surface of the fibre. Note that for type II fibres**,** Li(1) C_q = 572 kHz and Li(2) C_q = 284 kHz, meaning that there is a slight decrease in the quadrupolar coupling for Li(1) in addition to the appearance of the new site. This decrease is not significant enough to suggest a substantial structural change, however.

The variation in the lineshapes for type I and type II fibres (Fig. 4)) can be explained by analogy with bulk Li_3N[4]. Above 133 K all lines (but especially the Li(2) satellites) narrow with increasing temperature for both types of nanofibre. This can be interpreted in terms of intra-layer Li^+ diffusion between Li(2) sites. Subsequently, all the satellites (including the additional line observed for type II fibres) broaden again and eventually disappear into the baseline so that above 363 K the spectra contain only a narrow central line. This results from inter-layer diffusion *via* the exchange of Li(1) and Li(2) ions. At the lowest temperature, an additional pair of satellites is apparent for both types with a reduced quadrupolar coupling (C_q < 250 kHz) which has been attributed previously to the presence of defects[4].

For motional narrowing, the correlation time, τ_{NMR}, is related to the linewidth $\Delta\nu$ by the Bloembergen-Purcell-Pound (BPP) equation:

$$\Delta\nu(T)^2 \quad = \quad \Delta{\nu_0}^2 \frac{2}{\pi} tan^{-1} \ \{\Delta\nu(T)\pi\tau_{NMR}(T)\} \qquad (1)$$

where $\Delta\nu_0$ is the rigid-lattice linewidth. The experimental value of the regime 1 linewidth is close to the theoretical rigid-lattice linewidth calculated from the Van Vleck formula. For Li_3N τ_{NMR} corresponds to the mean jump rate of the Li(2) ions within the [Li_2N] planes and assuming a simple Arrhenius law allows measurement of the activation energy for diffusion, E_a (Supplementary Figure 8; Supplementary Table 3):

$$1/\tau_{NMR} = 1/\tau_0 \, exp \ (-E_a/kT) \qquad (2)$$

The diffusion coefficient, D, can be obtained using the Einstein relation:

$$D = \frac{fr^2}{g\tau_{NMR}} \qquad (3)$$

where r is the jump distance, f is a correlation factor which depends on the diffusion mechanism and g is a geometrical factor equal to 4 for planar diffusion.

Supplementary References

1. Stoeva, Z., Gomez, R., Gregory, D. H., Hix, G. B. and Titman, J. J. Evolution of structure, transport properties and magnetism in ternary lithium nitridometalates $Li_{3-x-y}M_xN$, M = Co, Ni, Cu. *Dalton Trans.* 3093–3097 (2004).
2. Domènech-Ferrer *et al.*, In situ Raman cell for high pressure and temperature studies of metal and complex hydrides. R. *Anal. Chem.*, **83**, 3199-3204 (2011).
3. Bohger, J.-P. O., Eßmann, R. R. and Jacobs, H. Infrared and Raman studies on the internal modes of lithium amide. *J. Mol. Struc.* **348**, 325-328 (1995).
4. Messer, R., Birli, H. and Differt, K. NMR Study of diffusion in Li_3N. *J. Phys. C Solid State* **14**, 2731–2746 (1981).
